# Emerging Trends in Endoscopic Bariatric Therapies: Personalization Through Genomics and Synergistic Pharmacotherapy

**DOI:** 10.3390/jcm14134681

**Published:** 2025-07-02

**Authors:** Wissam Ghusn, Annika Divakar, Yara Salameh, Kamal Abi Mosleh, Andrew C. Storm

**Affiliations:** 1 Department of Internal Medicine, Boston University, Boston, MA 02119, USA; wissamghusn7@gmail.com (W.G.); annika.divakar@bmc.org (A.D.); 2 Division of Gastroenterology and Hepatology, Mayo Clinic Hospital, Rochester, MN 55905, USA; salameh.yara@mayo.edu; 3 Department of Surgery, Mayo Clinic Hospital, Rochester, MN 55905, USA; abimoslehkamal@gmail.com

**Keywords:** endoscopic bariatric therapy, anti-obesity medication, GLP-1 receptor agonist, endoscopic sleeve gastroplasty, intragastric balloon, transoral outlet reduction, precision medicine, leptin–melanocortin pathway, obesity management

## Abstract

Obesity is a major global health challenge associated with significant metabolic and gastrointestinal comorbidities. While metabolic and bariatric surgery remains the gold standard for durable weight loss, the desire for additional options has fueled the development of endoscopic bariatric therapies (EBTs) as another tool for weight loss. This review examines established EBTs, including endoscopic sleeve gastroplasty (ESG), intragastric balloons (IGBs), and transoral outlet reduction (TORe), alongside emerging therapies such as duodenal mucosal resurfacing (DMR), incisionless anastomosis creation, and fully automated endoscopic gastric remodeling systems. ESG has demonstrated durable weight loss, favorable safety, and superior cost-effectiveness compared to pharmacotherapy alone, while combination strategies using EBTs and anti-obesity medications (AOMs), particularly GLP-1 receptor agonists, have resulted in greater total-body weight loss than either modality alone. Genetic variation, particularly within the leptin–melanocortin pathway, may predict response to endoscopic interventions and guide personalized treatment selection. Novel investigational procedures such as DMR, automated or robotic gastric remodeling, and magnetic or ultrasound-assisted gastric bypass show promising early results. Endoscopic therapies are poised to become increasingly central to the personalized, scalable management of obesity and related metabolic diseases.

## 1. Introduction

Obesity is the major global health issue of today, associated with a wide range of metabolic and cardiovascular comorbidities including type 2 diabetes (T2D), hypertension, dyslipidemia, certain cancers, and other cardiovascular diseases [1]. In recent years, the rising prevalence of obesity has placed significant strain on healthcare systems worldwide. By 2050, it is projected that 43.1 million children and adolescents (range: 37.2–47.4 million) and 213 million adults (range: 202–221 million) will be either overweight or living with obesity [2]. Obesity not only affects metabolic health but also has a profound impact on gastrointestinal (GI) physiology, contributing to conditions such as gastroesophageal reflux disease (GERD), metabolic dysfunction-associated steatotic liver disease (MASLD), and colorectal cancer [3].

Traditional management approaches, including lifestyle modification and pharmacotherapy (e.g., semaglutide and tirzepatide), have shown promising weight loss outcomes [4,5,6]. However, these interventions frequently fail to achieve durable weight loss or lasting metabolic benefits in individuals with severe obesity, especially once the medications are commonly discontinued [7,8]. In this context, bariatric interventions, particularly metabolic and bariatric surgeries (MBS) like Roux-en-Y gastric bypass (RYGB) and sleeve gastrectomy (SG), have emerged as the most effective treatments in terms of weight loss and resolution of the associated medical conditions [9,10,11]. Although MBS leads to significant weight loss, and metabolic and survival benefits, not all patients are suitable candidates due to their invasive nature, potential complications, and accessibility limitations [12,13,14].

Endoscopic bariatric therapies (EBTs) represent an increasingly valuable addition to the spectrum of obesity treatment modalities [15]. EBTs offer a less invasive option when compared to MBS and have shown promising results in promoting weight loss and improving metabolic health [16]. Their minimally invasive nature, along with a favorable safety profile, makes them particularly appealing for patients who may not be candidates for MBS or prefer to avoid surgery [15]. As an intermediate step between pharmacologic and surgical interventions, EBTs help broaden access to effective obesity care and have been shown to be more cost-effective than newer pharmacologic alternatives [17].

With the rising interest in personalized obesity management, there is growing emphasis on leveraging genomic insights and combining EBTs with anti-obesity medications (AOMs) to enhance outcomes. This review provides an in-depth exploration of current EBT modalities, their mechanisms, the role of genetic predictors in tailoring therapy, and the emerging synergy between EBTs and pharmacologic treatment.

## 2. Methods

This narrative review was informed by a structured literature search of PubMed, MEDLINE, Scopus, Embase, and Google Scholar, covering studies from database inception through April 2024. We used combinations of the following keywords: “endoscopic bariatric therapy”, “intragastric balloon”, “endoscopic sleeve gastroplasty”, “transoral outlet reduction”, “obesity pharmacotherapy”, “GLP-1 receptor agonist”, “semaglutide”, “tirzepatide”, and “genomics” or “genetic predictors”. Articles were limited to English-language publications involving adult human subjects. We prioritized randomized controlled trials, large cohort studies, systematic reviews, and meta-analyses, but also included relevant mechanistic or early-phase studies where applicable. Studies focusing exclusively on pediatric populations or animal models were excluded. Additional references were identified through manual review of bibliographies from key articles. This review aims to synthesize current evidence, highlight emerging trends, and identify future directions in the integration of endoscopic therapies with pharmacologic and genomic approaches to obesity care.

## 3. Overview of Endoscopic Bariatric Therapies (EBTs)

EBTs are minimally invasive endoluminal procedures designed to induce weight loss and improve metabolic parameters [18]. These interventions work through various mechanisms, including gastric restriction, delayed gastric emptying, altered nutrient absorption, and hormonal modulation [19]. EBTs are typically performed as outpatient procedures, offering reduced recovery time and fewer complications compared to traditional bariatric surgeries [20]. Indicated procedures with level 1 evidence for weight loss efficacy include the endoscopic sleeve gastroplasty (ESG), several intragastric balloons (IGBs), and the transoral reduction (TORe) procedure for revision post-RYGB surgery. Another FDA-approved EBT, aspiration therapy (e.g., AspireAssist), involves percutaneous gastrostomy-assisted aspiration of gastric contents post-meal. Although early studies demonstrated around 14–19% total-body weight loss (TBWL) sustained over four years [21], this modality has seen limited adoption and is no longer widely used; as such, it will not be discussed further in this review. The transpyloric shuttle is another FDA-approved device, designed to intermittently obstruct the pylorus and delay gastric emptying via a self-anchoring bulb connected by a flexible tether [22]. Despite early efficacy data showing modest weight loss [22], the device did not gain widespread clinical use and is likewise excluded from further discussion.

### 3.1. Approved and Clinically Available EBTs:

- Tissue Apposition Devices (TADs)

**Figure 1:**
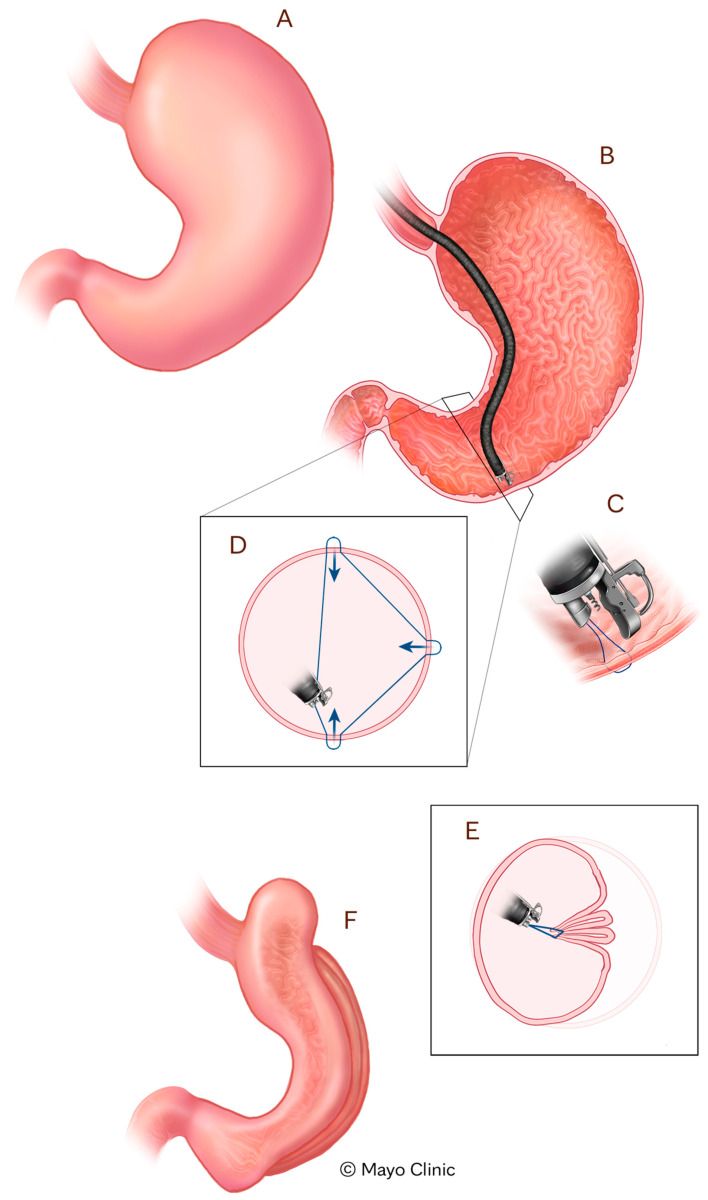
Endoscopic sleeve gastroplasty illustration. Endoscopic Sleeve Gastroplasty (ESG): (**A**) Normal stomach; (**B**) Endoscope insertion; (**C**) Suturing device deployment; (**D**) Stitch pattern placement; (**E**) Gastric plication along greater curvature; (**F**) Final sleeve configuration.

Tissue Apposition Devices (TADs) enable gastric remodeling via endoscopic suturing techniques designed to reduce stomach volume [23]. The most well-established example is ESG using the Apollo OverStitch Device (Boston Scientific, Marlborough, MA, USA), which mimics the restrictive mechanism of surgical SG while preserving the integrity of the stomach [23] (Figure 1). ESG has shown robust efficacy in clinical trials. In a multicenter randomized controlled trial, patients undergoing ESG combined with lifestyle modification achieved an average of 49.2% excess weight loss and 13.6% TBWL at 52 weeks, compared to only 3.2% and 0.8%, respectively, in the control group receiving lifestyle modification alone (*p* < 0.0001) [24]. Other studies have demonstrated similar efficacy in terms of achieved weight loss (e.g., 15–20% TBWL) and associated medical comorbidity remission [19,23]. The safety profile of this procedure has been shown to be favorable, with one multicenter study demonstrating a serious adverse event rate of only 2%, with none requiring surgical intervention and no mortalities [24]. In addition to its clinical efficacy, ESG has demonstrated superior cost-effectiveness compared to semaglutide, offering greater weight loss, modestly higher quality-adjusted life-years, and substantially lower total costs, underscoring its value as a durable and economically favorable intervention for individuals with class II obesity. In fact, in a 5-year economic evaluation, endoscopic sleeve gastroplasty (ESG) was more cost-effective than semaglutide for class II obesity, offering greater weight loss and USD 33,583 in cost savings, with semaglutide requiring a threefold price reduction to be cost-competitive [17]. These findings support ESG as a durable, safe, and cost-effective option for patients with class I or II obesity, offering both substantial weight loss and cardiometabolic benefit [17].

### 3.2. Space-Occupying Intragastric Devices (IGDs)

Intragastric devices (IGDs), particularly IGBs, are an example of space-occupying, volume-reduction devices, thereby promoting early satiety and supporting caloric restriction and weight loss [19]. Various types of IGBs are available, including single, dual, and adjustable balloons, each differing in volume, material, and filling medium (e.g., saline vs. gas) [25]. A comprehensive meta-analysis of 13 randomized controlled trials involving over 1000 patients demonstrated that IGBs are significantly more effective than lifestyle modification alone, achieving a difference in mean excess weight loss percentage (EWL%) of 17.98% and a TBWL of 4.4% compared to lifestyle interventions [26]. Several studies have shown that the average weight loss with IGBs ranges between 5 and 15% largely depending on the specific device being used [19]. Despite these benefits, durability of weight loss remains a concern, as weight recurrence is frequently observed after balloon removal [27]. Nonetheless, the reversible nature, relatively low complication rate, and effectiveness of IGBs position them as a valuable option for patients who have failed conservative measures and do not prefer or are ineligible for ESG or surgical intervention [28].

- Trans-Oral Reduction (TORe)

**Figure 2:**
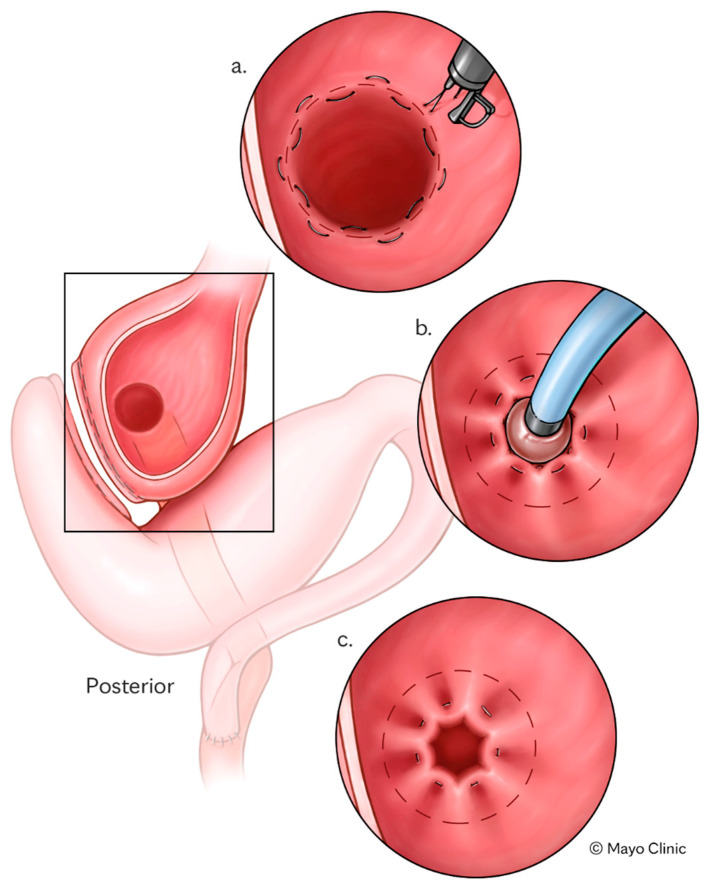
Trans-oral reduction illustration. TORe is performed using a purse-string suture pattern technique (**a**). A through-the-scope balloon is used to size the final anastomosis diameter upon tightening of the purse-string suture (**b**), which is secured over the balloon to secure the sutured construct and reduce the aperture of the gastrojejunal anastomosis (**c**).

TORe is a minimally invasive endoscopic intervention developed to address weight recurrence and dumping syndrome (DS) following RYGB, as well as other bariatric surgery anatomy [29,30] (Figure 2). After RYGB, the procedure involves endoscopic reduction of the diameter of the gastrojejunal anastomosis and/or gastric pouch, thereby delaying gastric emptying, enhancing satiety, and restoring the restrictive function of the bypass [31]. TORe is typically performed as an outpatient procedure using techniques including full-thickness suturing with argon plasma coagulation (APC) resurfacing [31]. TORe has demonstrated consistent safety and efficacy across multiple studies, achieving a TBWL of 8.5% at 1 year and maintaining clinically significant weight loss (≥5% TBWL) in over 60% of patients at 5 years [32].

In addition to weight recurrence after bariatric surgery, TORe has also been shown to effectively treat refractory DS by mitigating rapid gastric emptying. Clinical trials report a dramatic reduction in Sigstad scores and symptomatic resolution in up to 90% of patients within three months of the procedure [31,33]. Importantly, TORe is associated with a low incidence of serious adverse events, is repeatable if necessary, and is now considered a viable alternative to surgical revision, especially in patients with anticipated high peri-operative risk [34].

## 4. Personalized Approaches in Endoscopic Bariatric Therapies

### Genomic Influences on EBT Outcomes

Emerging evidence suggests that genetic variation significantly influences individual responses to EBTs. Among the key regulatory systems implicated is the leptin–melanocortin pathway (LMP), a central neuroendocrine axis involved in appetite and energy homeostasis [35]. In the postprandial state, leptin and insulin bind to receptors on pro-opiomelanocortin (POMC) neurons located in the arcuate nucleus of the hypothalamus. This stimulates the production of α-melanocyte-stimulating hormone (α-MSH). α-MSH then acts on melanocortin-4 receptors (MC4Rs) in the paraventricular nucleus (PVN), triggering satiety and reducing caloric intake. Concurrently, leptin signaling inhibits orexigenic neurons that co-express agouti-related peptide (AgRP) and neuropeptide Y (NPY), further suppressing hunger [35].

Variants in genes encoding key components of this pathway, such as LEPR, POMC, MC4R, and others, have been associated with altered satiety signaling, increased adiposity, and attenuated weight loss after metabolic interventions. These genetic differences may partially explain the interindividual variability observed in EBT outcomes. As precision medicine advances, incorporating genomic insights into clinical algorithms may help stratify patients more effectively. It also helps predict therapeutic response and tailor interventions such as EBTs for maximal metabolic benefit. Such an approach holds promise for improving long-term efficacy and reducing relapses in patients undergoing minimally invasive obesity treatment.

Studies have demonstrated that patients harboring single-nucleotide polymorphisms (SNPs) in the MC4R gene exhibit statistically significant differences in weight outcomes. In one study, researchers investigated the role of SNPs in modulating weight loss responses across different obesity treatment modalities, including dietary intervention, endoscopic procedures (e.g., IGB and POSE), and bariatric surgery. Among 474 patients stratified by treatment type, researchers identified 102 SNPs associated with baseline BMI and treatment-related weight loss outcomes. Notably, ten SNPs demonstrated a significant association with elevated BMI in the overall cohort. Variants in *PPARδ* and *ACSL5* showed consistent correlations with weight loss across all three modalities, while others, including *CASR* and *MC4R*, were uniquely associated with outcomes following endoscopic procedures [36]. This is just one example illustrating how genetic variation may influence outcomes following endoscopic weight loss interventions. Prior studies have also shown that individuals carrying heterozygous variants in the LMP experience significantly greater weight recurrence following TORe compared to non-carriers. In a case–control study, genotyped adults who underwent TORe were stratified by LMP variant status and followed over multiple time points, up to 12 months. Those with heterozygous variants consistently demonstrated lower TBWL at the specified time points post-procedure, with statistically significant differences between carriers and non-carriers [37]. Weight loss outcomes after TORe over 1 year have also been investigated. A study examined a larger cohort of patients after RYGB with or without heterozygous variants in the LMP and identified 34 variants across 21 genes, most commonly in PCSK1. Carriers of LMP variants experienced significantly less TBWL compared to non-carriers, 0.7% vs. 9.6% at 12 months (*p* < 0.01), despite undergoing the same TORe interventions. This pattern persisted across different TORe techniques, including tubular TORe and those involving argon plasma coagulation. These findings reinforce the potential impact of genetic variability on weight trajectory and suggest a role for incorporating genetic profiling into pre-procedural risk stratification (Table 1) [38,39].

These findings suggest that a patient’s genetic profile could be leveraged to predict responsiveness to EBT, enabling more personalized and effective treatment selection. By stratifying individuals based on their likelihood of weight loss or risk of weight recurrence, clinicians may better tailor interventions to maximize outcomes. Additionally, these insights support the rationale for a multimodal approach, combining endoscopic procedures with adjunctive pharmacotherapy, to enhance and sustain weight loss, particularly in patients with genetically mediated resistance.

However, it is important to note that these findings remain exploratory. The studies cited are primarily retrospective and observational, with small sample sizes and limited generalizability. No clinical protocols currently exist to guide EBT selection based on genetic profiles, and the majority of obesity remains polygenic, with modest, overlapping effects from individual SNPs. Although preliminary findings linking specific gene variants (e.g., MC4R, PCSK1, and LEPR) to EBT response are promising, routine genomic testing in clinical obesity practice remains limited. Key barriers include cost, lack of standardized panels, and the absence of prospective trials validating clinical decision-making based on genotype. However, as genotyping becomes more affordable and consumer-accessible, integration into pre-procedural evaluation may become feasible, particularly in academic or high-volume centers. Multi-omics approaches that combine genomics with behavioral, metabolic, and microbiome data could ultimately refine risk stratification and improve personalization of therapy. Future research should focus on operationalizing this model, including real-world implementation studies and cost-effectiveness analyses. In summary, genetic markers are an exciting frontier that could refine patient selection for EBTs. Equally important is the concept of combining therapies to maximize weight loss, which we explore next.

## 5. Synergy Between EBTs and AOMs

Recent advances in obesity management have underscored the potential of combining pharmacologic and endoscopic strategies to optimize weight loss outcomes and metabolic health. While both AOMs and EBTs independently induce significant weight reduction [40,41,42], emerging evidence suggests that their concurrent use may yield synergistic effects, improving both short- and long-term outcomes [43,44].

AOMs such as glucagon-like peptide-1 receptor agonists (GLP-1 RAs) enhance satiety, reduce caloric intake, and improve glycemic control [45]. When used adjunctively with EBTs, such as ESG or IGB placement, AOMs may reinforce behavioral and physiological adaptations initiated by the procedure, resulting in greater TBWL and improved durability of response (Table 2) [43,46].

### 5.1. ESG and AOMs

A multicenter US study evaluated 1506 patients who underwent ESG and analyzed the impact of AOM use on weight loss outcomes. Patients who started AOMs after ESG, especially after 12 months, achieved the greatest total-body weight loss, with over 20% TBWL at 24 months and nearly 90% maintaining at least 10% TBWL. In contrast, patients already on AOMs before ESG experienced the least weight loss. While GLP-1 receptor agonists appeared to result in greater weight loss at 18 and 24 months, the difference between drug classes was not statistically significant [47]. In another study, patients undergoing ESG with liraglutide achieved a 24.7% of TBWL at 12 months versus 20.2% with ESG alone [48]. A study by Jirapinyo et al. found that combining endoscopic gastric remodeling (EGR) with AOMs significantly improved weight loss outcomes compared to EGR alone. Patients who initiated AOMs within 6 months before or after EGR achieved greater total weight loss at 12 months (23.7 ± 4.6%) compared to those who underwent EGR monotherapy (17.3 ± 10.0%, *p* = 0.03). Notably, patients who had been on AOMs for more than 6 months prior to EGR had the lowest weight loss outcomes (12.0 ± 7.7%), suggesting that prolonged prior use of AOMs may reflect pharmacologic treatment resistance [49]. Another study evaluates the combined effect of ESG and AOM, specifically oral semaglutide, on weight loss and metabolic improvement. In this retrospective study of 18 patients, those who underwent ESG with AOM achieved significantly greater TBWL than those with ESG alone. At 6 months, TBWL was 20.27% vs. 15.32% (*p* = 0.02) in the combination group compared to ESG alone [50].

### 5.2. IGB and AOMs

In a study by Yilmaz et al., the efficacy of combining liraglutide with IGB therapy for weight loss was evaluated. In a cohort of 50 patients, those who received both IGB and liraglutide (Group B) achieved significantly greater reductions in weight, BMI, and percent body fat compared to those treated with IGB alone (Group A). At six months, median weight loss was 13.8 kg in Group B vs. 7.9 kg in Group A (*p* < 0.001), BMI reduction was 4.9 vs. 3.13 (*p* < 0.001), and body fat loss was also significantly higher [51]. In another retrospective study, 108 patients underwent IGB insertion, with 44 receiving adjunctive liraglutide. While patients treated with both IGB and liraglutide had greater mean weight loss at the time of balloon removal (18.5 ± 7.6 kg vs. 10.2 ± 6.7 kg, *p* < 0.0001), regression analyses revealed that those treated with IGB alone had significantly higher odds of sustained weight loss at 6 months post-removal (adjusted OR = 5.74, 95% CI: 1.79–188.42) [52]. Thus, despite initial enhanced weight loss, liraglutide did not improve long-term outcomes and may not offer added benefit when combined with IGB. Another trial assessing IGB combined with liraglutide showed a greater mean weight loss at balloon removal (18.5 kg vs. 10.2 kg; *p* < 0.0001) compared with IGB alone [46].

While GLP-1 receptor agonists represent the most established and studied pharmacologic adjuncts to EBTs, other anti-obesity agents, including phentermine/topiramate and bupropion/naltrexone, could theoretically be combined with endoscopic interventions. However, robust clinical data supporting these combinations are currently lacking. Moreover, tirzepatide, a dual GIP and GLP-1 receptor agonist with superior efficacy for weight loss, is an emerging agent of interest in this context. Although no clinical trials have yet evaluated tirzepatide in combination with EBTs, its potential for synergy with endoscopic approaches warrants future investigation. A forward-looking research agenda should prioritize evaluating such multimodal combinations to optimize long-term weight loss outcomes.

## 6. Procedural Options on the Horizon

### 6.1. Emerging Endoscopic Gastric Remodeling Devices

#### 6.1.1. Endomina (Endo Tool Therapeutics)

The Endomina system employs an over-the-scope triangulation platform that facilitates the creation of gastric plications along the greater curvature, effectively reducing stomach volume. This incisionless approach enables endoscopists to approximate and suture gastric tissue from within, mimicking the outcomes of laparoscopic interventions without external incisions. By decreasing gastric capacity, the procedure promotes early satiety, thereby aiding in weight loss [53]. Recent clinical trials have demonstrated its potential in achieving significant weight loss and improving metabolic parameters in patients with obesity. A randomized controlled trial evaluated the Endomina system (E-ESG) as an adjunct to lifestyle modification in patients with moderate obesity (BMI 30–40 kg/m^2^) [54]. At 6 months, the E-ESG group achieved a mean EWL of 38.6%, significantly higher than the 13.4% observed in the control group (*p* < 0.001). This weight loss was maintained at 12 months, with the E-ESG group reaching a mean EWL of 45.1% and a TBWL of 11.8%. Additionally, patients reported improved satiety and quality of life, with no severe procedure- or device-related adverse events noted [54]. In a multicenter prospective study, E-ESG demonstrated high technical success (100%), no serious adverse events, and meaningful weight loss (mean 48.5% EWL and 15.3% TBWL at 12 months) in patients with class I–II obesity. These findings confirm that ESG using the Endomina platform is a safe and effective large-scale therapeutic option for obesity [55]. The system’s ability to perform full-thickness sutures allows for durable tissue approximation, making it a promising tool in endoscopic bariatric interventions.

#### 6.1.2. Incisionless Operating Platform (IOP) by USGI Medical

Primary Obesity Surgery Endoluminal (POSE) is another minimally invasive endoscopic bariatric procedure not clinically available in the US that utilizes a specialized incisionless platform to create full-thickness plications in the gastric fundus and body, thereby reducing gastric volume and promoting early satiety [56]. A recent systematic review and meta-analysis including over 600 patients demonstrated that POSE is both safe and effective, with pooled mean EWL reaching nearly 49% and TBWL of approximately 13% at 12–15 months. Notably, weight loss outcomes at 1 year significantly favored POSE over control interventions in randomized controlled trials [56]. Other studies demonstrated a weight loss ranging between 10 and 20% [19,57]. The procedure also showed a low serious adverse event rate, with a mean procedural time under one hour, supporting its feasibility in clinical practice [56].

#### 6.1.3. EndoZip (Nitinotes Surgical)

The EndoZip™ system, not yet approved for use in the US, was developed by Nitinotes Surgical and is a fully automated, operator-independent endoscopic suturing platform designed to simplify the technical demands of endoluminal gastroplasty. By automating the suturing process, it enables consistent and reproducible gastric plications, reducing procedural variability and minimizing the learning curve associated with manual techniques. This minimally invasive approach aims to create durable gastric volume reduction [55]. The first-in-human study assessed the safety and feasibility of the fully automated EndoZip™ endoscopic suturing device for obesity treatment. In 11 patients with BMI 30–40 kg/m^2^, the procedure achieved 100% technical success, no serious adverse events, and a mean TBWL of 16.2% at six months. Most sutures remained intact at follow-up, supporting the device’s potential to simplify endoscopic gastroplasty and achieve meaningful weight loss [58]. In another prospective multicenter study evaluating the EndoZip for obesity treatment in patients with BMI 30–40 kg/m^2^, patients achieved a mean TBWL of 13.2%, with significant improvements in waist circumference, HbA1c, and quality of life at 12 months [59]. Serious adverse events occurred in 4.4% of cases [59]. The device was demonstrated to be safe, effective, and operator-friendly, offering a potential solution to reduce technical complexity in endoscopic bariatric procedures.

#### 6.1.4. SimpleStitch (EnVision Endoscopy)

SimpleStitch (SS) is a recently FDA cleared novel, circular endoscopic suturing device that simplifies the suturing process by reducing the number of accessory devices and procedural steps required [60]. In a randomized controlled ex vivo study, researchers compared SS, a novel circular endoscopic suturing device, to OverStitch (OS), the conventional suturing platform, for performing TORe [60]. The primary outcome, simulator score (i.e., points awarded for successful suturing), was significantly higher with SS (mean 179.3 ± 73.7) compared to OS (mean 99.8 ± 94.1; *p* = 0.049). The secondary outcome, mental workload assessed by the NASA Task Load Index (NASA-TLX; lower scores indicating less perceived workload), was significantly lower with SS (39.2 ± 15.9) versus OS (70.5 ± 22.1; *p* = 0.002). Among participants without prior suturing experience, SS led to markedly better simulator performance (133.8 ± 45.3 vs. 33.3 ± 20.7; *p* < 0.001) and lower workload scores (48.0 ± 14.4 vs. 84.3 ± 8.3; *p* < 0.001), while among experienced users, there was a non-significant trend favoring SS [60].

### 6.2. Duodenal Mucosal Resurfacing (DMR)

Duodenal mucosal resurfacing (DMR) is an endoscopic intervention that targets the proximal small intestine using hydrothermal ablation to induce mucosal regeneration [61]. This regeneration appears to restore insulin sensitivity and reset enteroendocrine signaling pathways implicated in glucose metabolism [62].

#### 6.2.1. Revita (Fractyl Health)

Revita is a hydrothermal DMR procedure that targets the duodenal mucosa to improve insulin sensitivity and glycemic control in patients with T2D [61]. In the REVITA-1 trial, patients with T2D undergoing a single DMR procedure demonstrated a durable reduction in HbA1c (−1.4%) over 24 months despite minimal changes in body weight and no increase in glucose-lowering medications [63]. Improvements were also observed in fasting plasma glucose, ALT, HDL cholesterol, and TG/HDL ratio, underscoring its systemic metabolic benefits. Notably, nearly 87% of patients maintained treatment durability in glycemic improvement at two years, and over half of patients reduced or maintained their baseline diabetes-medication use [63]. C-peptide and HOMA-IR levels also improved post-procedure, pointing to enhanced insulin sensitivity. While the modest TBWL (e.g., around 3 kg) suggests that DMR’s primary mechanism is not weight-dependent, its effects on liver enzymes and lipid profiles suggest a broader metabolic impact, particularly relevant in patients with insulin resistance and potential coexisting MASLD [63]. These findings support the promise of DMR as a safe, minimally invasive, adherence-independent metabolic therapy for T2D and related conditions [64]. Notably, Fractyl has shifted its focus from a primary diabetes treatment indication to targeting weight maintenance after discontinuation of GLP-1–based medications, as reflected in their ongoing IDE trial (REMAIN-1).

#### 6.2.2. Re-Cellularization via Electroporation Therapy (ReCET; Endogenex)

**Figure 3:**
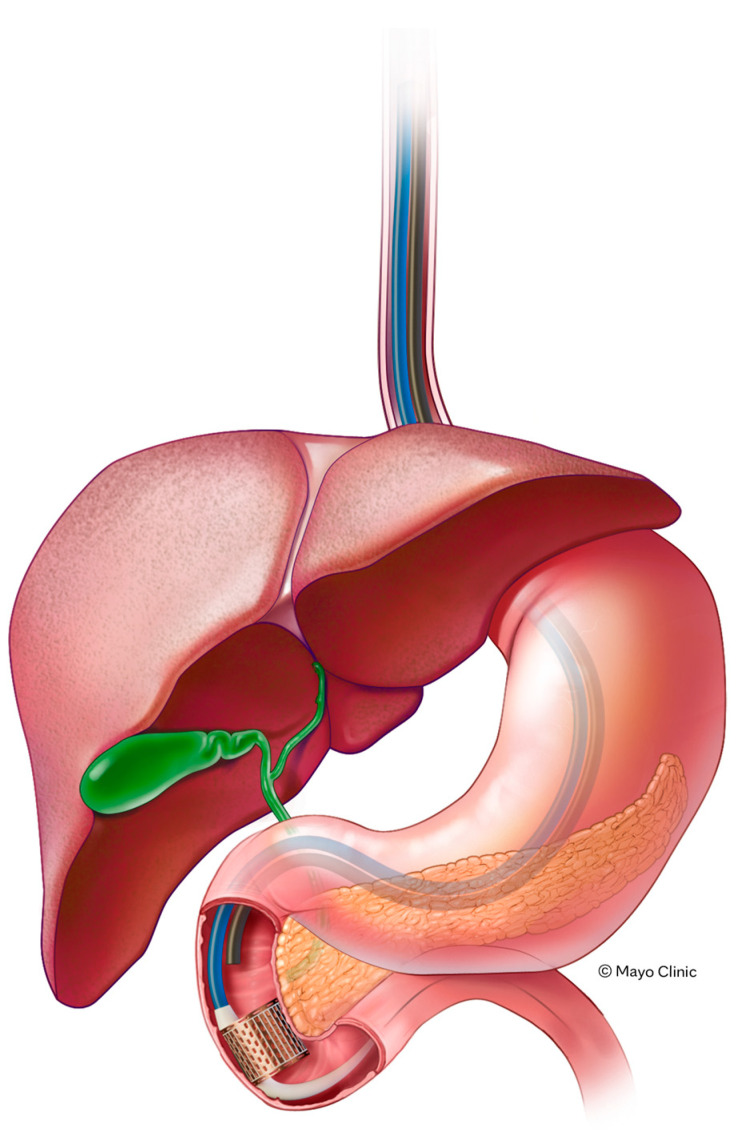
ReCET illustration. Novel resurfacing of the duodenum using pulsed electrical fields (ReCET) is performed for endoscopic treatment of type 2 diabetes.

ReCET employs pulsed electric fields, or electroporation, to treat the duodenal mucosa, promoting re-cellularization with metabolically active cells (Figure 3) [65,66]. This non-thermal approach aims to improve glycemic control in patients with T2D [67], with less risk than thermal-based alternative devices. In a first-in-human study evaluating the feasibility, safety, and efficacy of ReCET in patients with T2D on basal insulin, 14 patients underwent ReCET, followed by semaglutide initiation, with insulin discontinued after the procedure. At 12 months, 86% of patients remained off exogenous insulin, with significant improvements in glycemic control; no device-related severe adverse events were reported. These findings suggest that ReCET, combined with GLP-1 receptor agonist therapy, could offer a safe and effective alternative to insulin therapy for selected patients with T2D [65].

#### 6.2.3. The RESET Device from Morphic Medical

The Endoscopic Duodenal-Jejunal Bypass Liner, commercially known as EndoBarrier and more recently as RESET^®^ (Morphic Medical, Boston, MA, USA), is a 60 cm fluoropolymer sleeve with a crown-shaped nickel–titanium anchor at its proximal end. Inserted endoscopically into the proximal small intestine, it provides a temporary, reversible bypass of the duodenum and proximal jejunum, reducing nutrient absorption and mimicking the effects of a surgical bypass [68]. In an international registry of 1022 patients treated with the RESET device, mean HbA1c dropped from 8.5% to 7.2%, and mean weight decreased by 13.3 kg (from 106.2 kg to 92.9 kg). Systolic blood pressure declined by 12.9 mmHg, LDL cholesterol by 0.4 mmol/L, and 62% of patients reduced or stopped glucose-lowering medications; serious adverse events occurred in 4.2% of cases, including hepatic abscess in 1.1% [69]. In another study, RESET implantation led to a mean reduction in Apnea–Hypopnea Index from 18.9 to 9.7 events/hour (*p* < 0.001), allowing all 12 study participants to discontinue CPAP. At 12 months post-removal, 7 of 10 patients (70%) remained off CPAP [68]. Moreover, RESET^®^ is currently being evaluated in a pivotal, multicenter US clinical trial (STEP-1, NCT04101669) enrolling adults with obesity and type 2 diabetes to assess its safety and efficacy for improving glycemic control and promoting weight loss.

### 6.3. Endoscopic Anastomosis Creation Techniques

#### 6.3.1. Incisionless Anastomosis Devices (IADs)

Incisionless anastomosis devices (IADs), such as the incisionless magnetic anastomosis system (IMAS) or self-assembling magnets for endoscopy (SAMS), represent a novel endoscopic approach that mimics the effects of surgical bypass without the need for incisions [19]. These devices use self-assembling magnets to create a jejunoileal side-to-side anastomosis, effectively diverting nutrients to the distal ileum [19]. The procedure involves simultaneous enteroscopy and colonoscopy to position the magnets, which then compress the tissue to form the anastomosis. Over time, the magnets naturally pass in the stool, eliminating the need for removal [19]. The rerouted nutrient flow stimulates the release of gut hormones such as GLP-1 and PYY, enhancing satiety, improving glycemic control, and promoting weight loss [70]. In a small pilot study, IMAS led to a TBWL of approximately 14.6% and a reduction in HbA1c by 1.9% in patients with diabetes at 12 months [70]. These promising early outcomes support the potential of IADs in obesity and metabolic disease management, although larger studies are needed to validate their long-term efficacy and safety.

#### 6.3.2. Endoscopic Ultrasound (EUS)-Guided Anastomosis

A preclinical study assessed the feasibility of a novel endoscopic approach, Ultrasound-Assisted Endoscopic Gastric Bypass (USA-EGB), in live swine models [71]. The procedure involved four sequential steps: balloon-assisted enteroscopy to determine bypass limb length, EUS-guided creation of a gastroenteric anastomosis using a lumen-apposing metal stent (LAMS), endoscopic closure of the pylorus, and gastric volume reduction via transmural suturing. Complete gastrointestinal bypass with concurrent gastric restriction was achieved in all three animals without major complications, with a mean total procedure time of 131 min. These findings suggest that USA-EGB is technically feasible, potentially reversible, and may serve as a minimally invasive alternative to surgical gastric bypass pending further clinical evaluation [71]. While EUS-GJ has been extensively studied for gastric outlet obstruction, its application for primary weight loss remains investigational and untested in human trials.

## 7. Challenges and Future Directions

While EBTs represent an exciting and rapidly advancing frontier in obesity management, several real-world barriers may limit their widespread adoption. Chief among these is inconsistent insurance coverage [72]. In the United States and many other healthcare systems, reimbursement for procedures such as ESG and IGBs remains limited, often requiring patients to pay out of pocket [73]. This financial barrier restricts access to a narrow subset of patients, undermining the equity and scalability of these interventions.

Beyond coverage, the magnitude and durability of weight loss from EBTs must be interpreted within context. While procedures like ESG can achieve 15–20% total-body weight loss and TORe is effective for post-surgical weight recurrence, most endoscopic therapies yield less weight loss than bariatric surgery, which typically exceeds 25–30% TBWL [74]. Furthermore, durability beyond 2–3 years is not yet well-established for many EBTs, with limited five-year data currently available only for ESG.

Patient selection remains another challenge. Although emerging genomic tools hold promise for personalized stratification, clinical success with EBTs is also heavily influenced by behavioral, psychological, and dietary factors. Identifying the optimal procedure for each patient remains complex; for instance, those with uncontrolled binge eating may struggle with volume-based therapies like IGBs, while patients unable to sustain lifestyle changes may regain weight even after technically successful ESG. Thus, a comprehensive multidisciplinary evaluation, including nutritional, psychological, and medical assessment, is essential to support long-term efficacy.

Operator experience and procedural training also play a critical role in outcomes. EBTs, while less invasive, are technically demanding and require a learning curve. Complication rates and procedural efficiency are optimal in high-volume centers with trained endoscopists. As new techniques such as EUS-guided bypass and magnet-based interventions enter clinical trials, it will be essential to establish standardized protocols and safety profiles.

Finally, regulatory clarity is needed. Several promising devices discussed in this review, including DMR, IADs, and EUS-guided gastrojejunal bypass, are not yet FDA-approved and remain investigational. For clinicians and policymakers, distinguishing between commercially available tools and experimental technologies will be important in guiding adoption, patient counseling, and research prioritization.

## 8. Conclusions

EBTs represent a dynamic and rapidly evolving field that fills a critical gap between lifestyle interventions and bariatric surgery. As the number and diversity of EBTs continue to expand, tailoring procedural selection to individual patient profiles becomes increasingly important. This review has highlighted how clinical, anatomical, metabolic, and emerging genetic factors may influence response to specific interventions. For instance, ESG and intragastric balloons have demonstrated synergy with GLP-1 receptor agonists, supporting combination strategies in select patients. Meanwhile, early genetic studies suggest that response to certain EBTs may be partially mediated by variants in pathways such as leptin–melanocortin signaling, though clinical application remains premature. While newer technologies such as DMR, EUS-guided bypass, and incisionless anastomoses show promise, they remain investigational and require further study before integration into practice.

Moving forward, procedural selection should be guided by a holistic framework that incorporates not only BMI and comorbidities, but also behavioral readiness, access to pharmacotherapy, anatomical considerations, and, eventually, molecular markers. A multidisciplinary approach that integrates endoscopic expertise, metabolic management, and individualized risk assessment will be essential for optimizing outcomes and expanding access to effective obesity treatment.

## Figures and Tables

**Table 1:** Impact of leptin–melanocortin pathway variants on weight loss outcomes after transoral outlet reduction (TORe).

Reference	Author	Population	Design	Participants Number	TBWL (%) at 12 Months
[37]	Gala et al.	Patient with leptin–melanocortin pathway variant carriers vs. non carriers after TORe	Case–control	4 carriers, 10 controls	−1.5 vs. −12.3
[38]	Gala et al.	Patient with leptin–melanocortin pathway variant carriers vs. non carriers after TORe	Cohort	22 carriers, 32 controls	−0.68 vs. −9.6

**Table 2:** Weight loss outcomes with endoscopic bariatric therapies combined with anti-obesity medications (AOMs).

Reference	Author	Study/Procedure	Design	Participants Number	Total-Body Weight Loss
[47]	Gala et al.	ESG + AOM	Retrospective	1506	With GLP1-RA: −16.7% vs. other AOM: −14.2%
[48]	Badurdeen et al.	ESG + liraglutide	Prospective	66	With AOM: −24.7% vs. without: −20.2%
[49]	Jirapinyo et al.	EGR + AOM	Retrospective	208	With GLP-1RA: −23.7% vs. no AOM: −17.3%
[50]	Chung et al.	ESG + oral semaglutide	Retrospective	18	With semaglutide: −20.27% vs. without: −15.32%
[51]	Yilmaz et al.	IGB + liraglutide	Prospective	50	With liraglutide: −13.8 kg vs. without: −7.9 kg
[52]	Mosli et al.	IGB + liraglutide	Retrospective	108	With liraglutide: −18.5 kg vs. without: −10.2 kg

ESG: Endoscopic Sleeve Gastroplasty; AOM: Antiobesity Medications; GLP-1RA: Glucagon-like protein 1 receptor agonist; IGB: Intra-gastric balloon.
